# The Effect of Hepatosteatosis on Response to Antiviral Treatment in Patients with Chronic Hepatitis B: A Meta-Analysis

**DOI:** 10.1155/2017/1096406

**Published:** 2017-03-21

**Authors:** Yongfen Zhu, Qiao Yang, Fangfang Lv, Yunsong Yu

**Affiliations:** Department of Hepatology and Infection, Sir Run Run Shaw Hospital, Zhejiang University, Hangzhou, China

## Abstract

*Background.* This study is to systematically analyze the effects of hepatosteatosis on the response to antiviral treatment in patients with chronic hepatitis B (CHB) and hepatosteatosis. *Methods.* Systematic search was performed in PubMed, Embase, Web of Science, Elsevier, and the Chinese BioMedical literature databases for relevant studies published until February 2016. Treatment outcomes were compared between patients with CHB plus concomitant hepatosteatosis and those without hepatosteatosis. *Results.* A total of 8 prospective cohort studies (399 patients with CHB plus hepatosteatosis and 688 patients with only CHB) were included. Biochemical and virological response at both 48 and 96 weeks were significantly lower in patients with CHB plus hepatosteatosis as compared to that in patients with only CHB. Subgroup analysis based on methods used for diagnosis of hepatosteatosis and treatment regimens showed that when hepatosteatosis was diagnosed on Doppler ultrasound and treated with nucleotide analogues, patients with CHB plus hepatosteatosis showed lower biochemical (62.7% versus 75.8%, *P* = 0.002) and virological response (66.2% versus 72.3%, *P* = 0.006) as compared to that in patients with CHB. *Conclusion.* Hepatosteatosis lowers the efficacy of antiviral treatment in patients with CHB, especially when hepatosteatosis was diagnosed on ultrasound findings and treated with nucleotide analogues.

## 1. Introduction

Hepatitis B virus (HBV) infection is one of the main causes of chronic liver disease and accounts for more than 350 million people with chronic hepatitis B (CHB) worldwide. Patients with persistent HBV infection show wide variability in clinicopathological manifestations ranging from minimal histological changes to liver cirrhosis, hepatocellular carcinoma (HCC), or even acute-on-chronic liver failure [1, 2]. Nonalcoholic fatty liver disease (NAFLD) is characterized by fat deposition in hepatocytes and is associated with liver damage ranging from simple steatosis to liver fibrosis, cirrhosis, and HCC [3]. With socioeconomic development and lifestyle changes, NAFLD is increasingly being recognized as a public health concern with estimates of prevalence ranging from 5 to 40% in the general population [4]. It is now becoming the most common liver disease in the general population worldwide [5].

An increase in patients who have CHB with concomitant NAFLD has been reported [6, 7]. Since the pathogenesis of CHB and NAFLD is complex, they may affect each other. Thus, coexistence of CHB and NAFLD may exhibit novel pathophysiological characteristics.

Early stage of NAFLD is defined as the presence of steatosis in more than 5% of hepatocytes [8]. HBV X protein is reported to induce hepatic steatosis by enhancing the expression of liver fatty acid binding proteins [9]. Recent studies have revealed a relatively common finding of steatosis in CHB patients; further, the incidence of steatosis is much higher in patients with CHB as compared to that in the general population, implying its role in CHB [8] Moreover, both HBV infection and steatosis can lead to necroinflammation in the liver. Thus, it is difficult to distinguish the cause of hepatic necroinflammation. Therefore, the presence of hepatic steatosis may adversely affect the efficacy of antiviral therapy [10, 11].

Several recent clinical studies have investigated the impact of superimposed hepatosteatosis on the response to antiviral treatment in patients with CHB; however, the results have been inconsistent. We conducted a meta-analysis to systematically analyze the effects of hepatosteatosis on the response to antiviral treatment in patients with CHB and hepatosteatosis.

## 2. Methods

### 2.1. Search Strategy and Study Selection

Systematic search was performed on PubMed, Embase, Web of Science, Elsevier, and the Chinese BioMedical literature databases for articles published as of February 2016. The following keywords were used during the search: “chronic hepatitis B” or “inflammation of liver caused by hepatitis B virus”; “hepatic steatosis” or “hepatosteatosis” or “fatty liver” or “NAFLD” or “NASH”; and “antiviral therapy” or “nucleotide analogue” or “peginterferon alfa” or “standard interferon alfa.” Titles and abstracts of retrieved studies were first scanned, and the full texts of potential eligible studies were reviewed. The retrieved studies were carefully examined to exclude potential duplicates or papers with overlapping data. Only full-text publications compared the response to antiviral treatment in patients with CHB and concomitant hepatosteatosis with those in CHB without hepatosteatosis. Studies that were not published as full reports, such as conference abstracts and letters to the editors, were excluded. Reports cited in the references and relevant reviews were also manually searched to include potentially missed studies.

### 2.2. Data Extraction and Outcome Definitions

Data was extracted independently by two authors; any discrepancies were resolved by consensus amongst the authors. The following information was extracted from each trial: publication details (title, the first author, and place of the study conducted), study design (inclusion and exclusion criteria), participant details (the numbers of patients enrolled, age), intervention details (including type and dose of interferon, nucleotide analogue, and mode of administration), duration of treatment and follow-up, and outcomes. The outcomes included biochemical response (time taken for the serum levels of aminotransferase to return to normal), virological response (time taken for the HBV DNA to become undetectable), and serological response (time taken for the disappearance of HBeAg and the appearance of anti-HBe).

Quality assessment of the included studies was done by two authors using an improved Newcastle-Ottawa Scale [12]. Studies which scored ≥9 points were deemed to be of high quality; those with 5–8 points and <5 points were deemed to be of moderate and low quality, respectively.

### 2.3. Statistical Analysis

Heterogeneity between individual studies was assessed by *I*^2^ test. A value greater than 75% was considered indicative of a substantial heterogeneity; that between 50% and 75% was considered indicative of moderate heterogeneity; that between 25% and 50% was considered indicative of mild heterogeneity; and a value < 25% was considered indicative of absence of heterogeneity. A random effects model was used in the event of significant heterogeneity; a fixed effects model was used otherwise. The impact of publication bias was assessed using the Egger regression asymmetry test. Funnel plots were constructed if a sufficient number of studies with low heterogeneity were available. A *P* value < 0.05 was considered statistically significant. STATA 11.0 software (Stat Corporation, College Station, Texas, USA) was used for all analyses.

## 3. Results

### 3.1. Search Results and the Characteristics of the Included Studies

**Figure 1:**
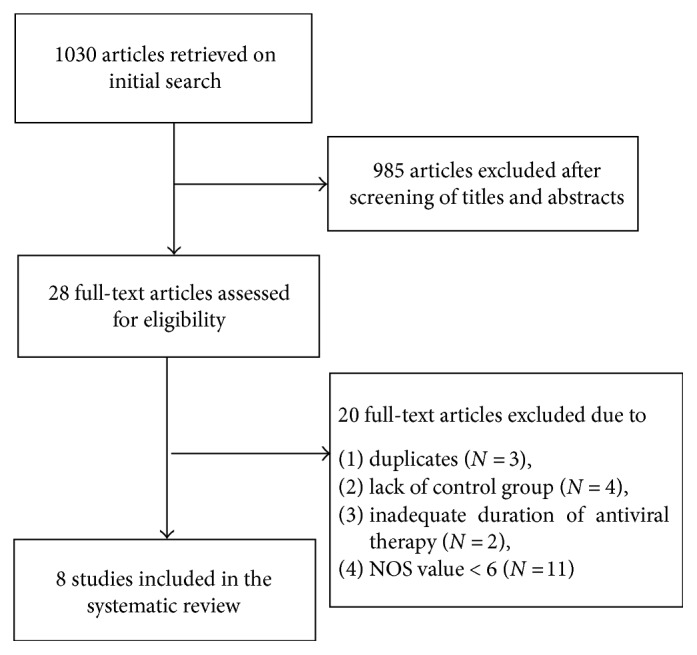
Flow chart showing study selection for the meta-analysis.

A total of 1030 articles were retrieved on initial literature search, of which 28 were deemed to be potentially relevant on a review of titles and abstracts. After a careful review of the 28 full-text articles, 3 were excluded owing to data duplication; four were excluded due to the lack of a control group; two were excluded because of inadequate duration of antiviral treatment; 11 were excluded as the NOS scores were <6. Finally, a total of 8 articles were included in the meta-analysis [13–20] (Figure 1).

The general information of the included studies is shown in Table 1. Amongst these studies, 2 were conducted in Turkey [13, 16] and 6 in China [14, 15, 17–20]. Five studies were published in English; three were in Chinese. In four studies, patients were treated with only interferon-alpha [13, 17, 19, 20]; in three studies, patients were treated only with nucleoside analogues [14, 15, 18]; and in one study, the patients were treated with interferon-alpha in combination with nucleoside analogues [19]. Four trials are comprised of 48-week interferon-alpha treatment; two were with 48-week follow-up [19, 20] and the other two were with 96-week follow-up [13, 17]. One trial is comprised of 48-week treatment with interferon-alpha combined with nucleoside analogues and 48-week follow-up [16]. Three trials are comprised of 96-week treatment with nucleoside analogues and 96-week follow-up [14, 15, 18]. Four studies were prospective cohort studies [13, 17–19] while the other four studies were retrospective cohort studies [14–16, 20].

Baseline data, including alanine aminotransferase (ALT), aspartate aminotransferase (AST), HBV DNA level, and the percentage of patients who were HBeAg positive, are shown in Table 2.

### 3.2. Biochemical, Virological, and Serological Responses at 48 Weeks

**Figure 2:**
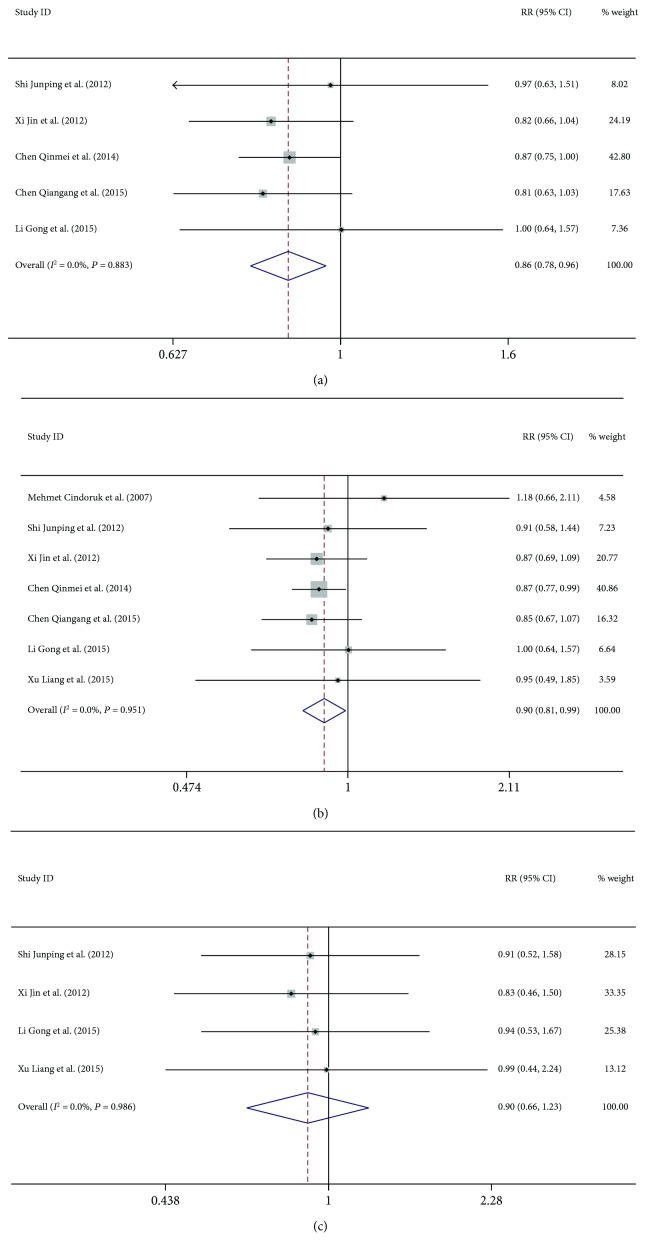
Forest plot of (a) biochemical, (b) virological, and (c) serological responses at 48 weeks.

Five trials with a combined study population of 325 patients with CHB plus steatosis and 530 patients with only CHB reported data on biochemical response at 48 weeks [14, 15, 17–19]. The result is shown in Figure 2(a). No substantial heterogeneity was observed amongst these studies (*I*^2^ = 0%, *P* = 0.083), and a fixed effects model was used for the analysis. Patients with CHB plus steatosis showed a lower rate of biochemical response at 48 weeks as compared to that in patients with only CHB (59.7% versus 69.6%; risk ratio (RR) = 0.86, 95% CI 0.78–0.96, *P* = 0.007).

Seven studies had reported data on virological response at 48 weeks [13–16, 18, 19]. No substantial heterogeneity was observed (*I*^2^ = 0%, *P* = 0.95), and the fixed effects model was used. Patients with CHB and steatosis showed a lower rate of virological response at 48 weeks as compared to that observed in patients with only CHB (58.7% versus 65.3%, RR = 0.90, 95% CI 0.81–0.99, *P* = 0.03, Figure 2(b)).

Four trials addressed the serological response to antiviral treatment at 48 weeks [17–20]. No statistically significant heterogeneity was observed amongst these studies (*I*^2^ = 0%, *P* = 0.99). The estimated pooled RR value showed no significant between-groups difference (27.6% versus 29.7%, RR = 0.90, 95% CI 0.66–1.23, *P* = 0.504, Figure 2(c)).

### 3.3. Biochemical, Virological, and Serological Responses at 96 Weeks

**Figure 3:**
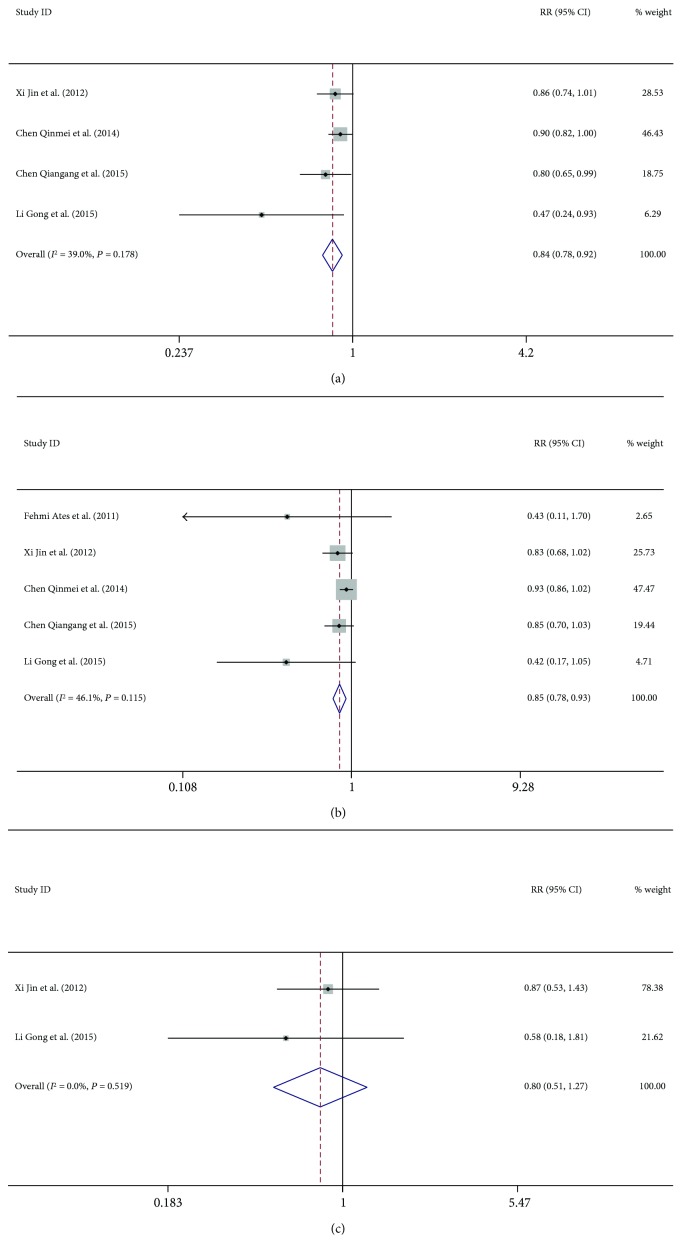
Forest plot of (a) biochemical, (b) virological, and (c) serological responses at 96 weeks.

Five studies reported data on biochemical response at 96 weeks, which showed no heterogeneity (*P* = 0.178, *I*^2^ = 39%) [14, 15, 17, 18]. The pooled RR showed a significantly lower sustained biochemical response in patients with CHB and steatosis as compared to that in patients with only CHB (71.2% versus 86.6%, RR = 0.85, 95% CI 0.78–0.93, *P* = 0.000, Figure 3(a)).

Data on virological response at 96 weeks was available for 4 trials [13–15, 17, 18]. The pooled RR showed a significantly lower virological response in patients with CHB and steatosis as compared to that in patients with only CHB (67.3% versus 75.2%, RR = 0.84, 95% CI 0.78–0.92, *P* = 0.000). No heterogeneity was observed amongst these studies (*I*^2^ = 46.1%, *P* = 0.12) (Figure 3(b)).

Only two studies reported data on serological response at 96 weeks [17, 18]. The fixed effects model was used for the analysis owing to no substantial heterogeneity (*P* = 0.51, *I*^2^ = 0.0%). No significant between-groups difference was observed with respect to sustained serological response (22.9% versus 28.5%, RR = 0.80, 95% CI 0.51–1.27, *P* = 0.35, Figure 3(c)).

### 3.4. Subgroup Analysis Based on the Method Used for the Diagnosis of Hepatosteatosis and Treatment Regimens

**Figure 4:**
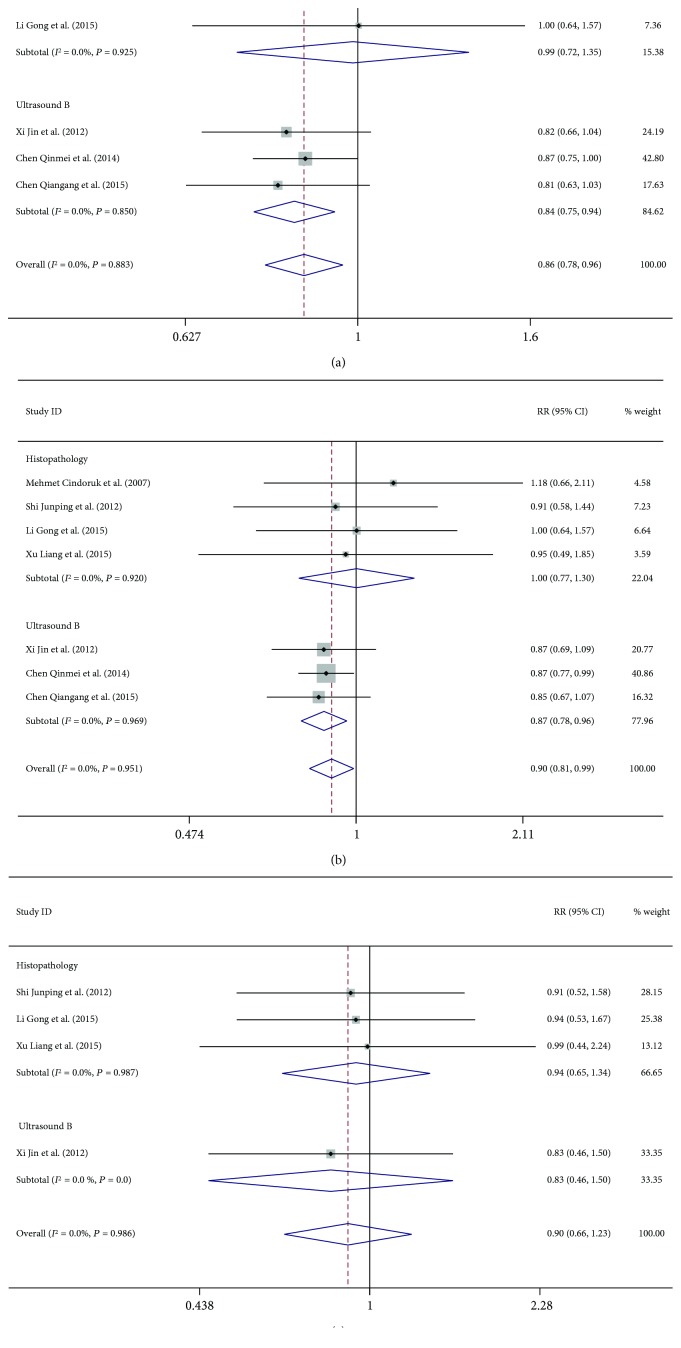
Subgroup analysis of (a) biochemical, (b) virological, and (c) serological responses at 48 weeks.

Next subgroup analyses on diagnosis methods and treatment regimens were performed using the outcome of virological response at 48 weeks. We found that patients diagnosed by liver biopsy were all treated with interferon, and patients diagnosed by Doppler ultrasound were all treated with nucleotide analogues. Thus, subgroup analysis was performed according to the methods used for the diagnosis of hepatosteatosis and treatment regimens: Doppler ultrasound and nucleotide analogues [14, 15, 18] or liver biopsy and interferon [13, 16, 17, 19, 20]. Subgroup analysis showed that if hepatosteatosis was diagnosed by liver biopsy and treated with interferon, there was no significant difference in biochemical response (47.6% versus 48.3%, *P* = 0.934), virological response (42.5% versus 42.3%, *P* = 0.987), or serological response (34.5% versus 37.1%, *P* = 0.718). However, if hepatosteatosis was diagnosed by Doppler ultrasound and treated with nucleotide analogues, significant differences were observed in the biochemical (62.7% versus 75.8%, *P* = 0.002), virological (66.2% versus 72.3%, *P* = 0.006), and serological responses (18.5% versus 22.3%, *P* = 0.533) between the two groups (Table 3, Figures 4(a), 4(b), and 4(c)).

### 3.5. Risk of Bias

**Figure 5:**
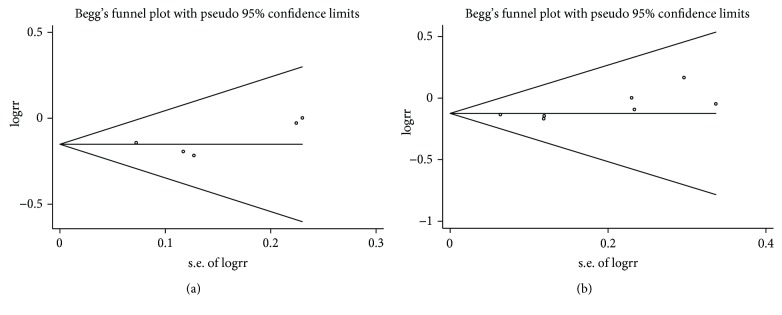
Begg's funnel plot of publication bias based on (a) biochemical and (b) virological responses at 48 weeks.

Publication bias of the included articles was performed using Begg's and Egger's tests based on outcomes of biochemical response and virological response at 48 weeks. For biochemical response at 48 weeks, no publication bias was detected (Begg's test, *P* = 0.46; Egger's test, *P* = 0.41; Figure 5(a)). For virological response at 48 weeks, no publication bias was detected (Begg's test, *P* = 0.07; Egger's test, *P* = 0.08; Figure 5(b)).

## 4. Discussion

Due to increase in consumption of fat-rich diet coupled with sedentary lifestyle, the incidence of NAFLD is increasing [8]. Thus, the frequency of patients with CHB and concomitant NAFLD is also increasing. Definitive evidence of the effect of hepatosteatosis on the efficacy of antiviral therapy in patients with CHB is yet to be achieved. In the present meta-analysis, 8 cohort studies, published between 2002 and 2013, with a combined subject population of 399 patients with CHB plus hepatosteatosis and 688 patients with only CHB, were included. All patients received interferon or nucleotide analogues for >1 year. The meta-analysis showed significantly lower biochemical and virological responses in CHB patients with hepatosteatosis at both 48 and 96 weeks, as compared to those in patients with only CHB. Our data suggests that hepatosteatosis decreased the response to antiviral therapy in CHB patients.

Response to anti-HBV therapy is dependent on a number of variables. Of these, baseline HBeAg status, HBV DNA level, and HBV genotype are the most important predictors [21, 22]. The studies included in the present meta-analysis did not report HBV genotypes. Except for the study conducted by Shi et al. [19], no significant difference was observed in baseline HBeAg status and HBV DNA level between the two groups. Contrary to the general belief that high HBV DNA level is a predictor of poor response to antiviral treatment [23], Shi et al. [19] found that the patients with CHB and hepatosteatosis had lower HBV DNA levels than the patients with CHB alone. This indicates that baseline HBV DNA level may not contribute to the response to treatment.

Both CHB and NAFLD cause chronic inflammation in the liver, which manifests as an increase in ALT level. It is hard to differentiate the cause of these two inflammatory diseases based on clinical presentation, though the two can be differentiated by histopathological examination. CHB patients show different degree of inflammatory in the portal area and surroundings, and inflammatory cells aggregate in the portal area to enlarge it; patients with NAFLD show lobular inflammatory and hepatosteatosis with low inflammatory infiltrate in the portal area, and the lobular inflammatory cell infiltration positively correlates with liver damage [24, 25]. Confirming the location of liver inflammation on biopsy can differentiate the cause of chronic inflammation.

Further subgroup analysis was performed based on the diagnostic methods and treatment regimens. It is interesting that in the included papers, when the patients were diagnosed by Doppler ultrasound, they were treated with nucleotide analogue; while when they were diagnosed by liver biopsy, they were treated with interferon. Our data showed that when hepatosteatosis was diagnosed by Doppler ultrasound and treated with nucleotide analogues, patients with CHB and hepatosteatosis showed lower biochemical and virological responses compared to patients with only CHB. Such a difference was not noted between the two groups when the diagnosis of hepatosteatosis was based on biopsy and treated with interferon.

Although Doppler ultrasound has some limitations such as low sensitivity and high subjectivity in imaging diagnosis of hepatosteatosis, it has been widely applied in clinic because of noninvasion, low expense, and easy access especially in China [26]. In all the included papers, the patients with CHB and hepatosteatosis started to receive antiviral treatment when their ALT levels and HBV DNA levels were abnormal according to the standard treatment criteria for CHB [27]. Moreover, all these studies applied Doppler ultrasound for the diagnosis of hepatosteatosis because of the abovementioned advantages. Why lower efficacy of antiviral therapy was observed in the patients with CHB and hepatosteatosis than in patients with CHB alone and the underlying mechanism remains unclear. Some researchers speculated that the ALT abnormity because of hepatosteatosis-induced liver chronic inflammatory was always misdiagnosed as the activation of HBV, thus leading to early antiviral treatment, which may result in poor response to antiviral treatments in CHB patients with hepatosteatosis [18]. Our data were consistent with this speculation. This may be because experienced pathologists can distinguish CHB patients from CHB and NAFLD patients by biopsy, while doctors cannot differentiate CHB and NAFLD patients by Doppler ultrasound, which leads to the early antiviral treatment and thus poor response to treatments. Further studies are needed to confirm this since no detailed information for diagnosis was provided in the included studies. Anyway, our data suggest that, for CHB patients with hepatosteatosis, when the disease cause cannot be confirmed, biopsy can help us to confirm the cause and thus improve the response to antiviral treatments.

There are several limitations in this meta-analysis that ought to be highlighted. First, most studies included in the meta-analysis were retrospective, single-center studies. Secondly, the sample size in some of the studies was small. Both of these factors could have introduced an element of bias and affect the results of the meta-analysis. More prospective, multicenter observational studies are required to confirm our findings.

## 5. Conclusion

This meta-analysis indicates that hepatosteatosis in patients with CHB is associated with decreased response to antiviral treatment, especially when hepatosteatosis was diagnosed based on ultrasound findings and treated with nucleotide analogues.

## Conflicts of Interest

The authors declare that they have no conflict of interest.

## Figures and Tables

**Table 1:** Characteristics of the studies included in this meta-analysis.

Study	Year	Location	Sample size	Number included in the analysis	Age-matched	Sex-matched	Follow-up (weeks)	Therapy regimen	Therapy period	Diagnosis method	Quality scores
Cindoruk et al. [16]	2002–2006	Turkey	140	98	Yes	Yes	48	Peg-IFN alfa-2a 180 *μ*g/week or Peg-IFN alfa-2b 80 *μ*g/week and LAM 100 mg/d	48	Histopathology	7
Ates et al. [13]	2006–2009	Turkey	84	84	No	Yes	96	Peg-IFN alfa-2a 180 *μ*g/week or Peg-IFN alfa-2b 80 *μ*g/week	48	Histopathology	6
Shi et al. [19]	2007-2008	China	120	96	No	Yes	48	Peg-IFN alfa-2a 180 *μ*g/week or Peg-IFN alfa-2b 80 *μ*g/week	48	Histopathology	6
Jin et al. [18]	2007–2009	China	267	213	Yes	Yes	96	ETV 0.5 mg/d	96	Ultrasound	9
Chen et al. [14]	2007–2009	China	332	316	Yes	Yes	96	LAM 100 mg/d and ADV 10 mg/d or ETV 0.5 mg/d	96	Ultrasound	8
Chen et al. [15]	2006–2012	China	141	141	Yes	Yes	96	ETV 0.5 mg/d	96	Ultrasound	7
Gong et al. [17]	2010–2013	China	97	89	No	Yes	96	Peg-IFN alfa-2a 180 *μ*g/week	48	Histopathology	6
Xu et al. [20]	2005–2009	China	50	50	Yes	No	48	Peg-IFN alfa-2a 180 *μ*g/week	48	Histopathology	6

**Table 2:** Baseline biochemical levels in the trials included in the meta-analysis.

	Biochemical baseline (IU/L)		Baseline HBV DNA level (log10 copies/mL)		Patients with HBeAg(+) (%)	
Study	With hepatosteatosis	Without hepatosteatosis	With hepatosteatosis	Without hepatosteatosis	With hepatosteatosis	Without hepatosteatosis
Cindoruk et al. [16]	ALT 105 ± 75^▲^	ALT 155 ± 39	NA	NA	HBeAg(+) 68.8%	HBeAg(+) 70.7%
	AST 111 ± 64^▲^	AST 132 ± 49				
Ates et al. [13]	ALT 128.3 ± 18.9^▲▲^	ALT 139.2 ± 52.5	3.72 ± 3.38	3.74 ± 4.12	HBeAg(+) 21.1%	HBeAg(+) 26.2%
	AST 90.7 ± 34.8	AST 107 ± 40.3				
Shi et al. [19]	ALT 142.5 ± 84.1	ALT 177.1 ± 138.3	6.96 ± 1.27^▲^	7.54 ± 1.28	HBeAg(+) 100%	HBeAg(+) 100%
	AST 72.9 ± 35.3^▲^	AST 99.5 ± 72.3				
Jin et al. [18]	ALT 171.68 ± 46.23	ALT 159.18 ± 45.12	6.69 (5.94–7.50)	6.65 (5.18–7.51)	HBeAg(+) 58.5%	HBeAg(+) 64.2%
	AST 59.66 ± 13.81	AST 56.63 ± 13.13				
Chen et al. [14]	ALT 250.5 ± 145.2	ALT 286.0 ± 210.2	6.8 ± 1.2	6.9 ± 1.1	HBeAg(+) 100%	HBeAg(+) 100%
	AST 149.1 ± 101.8	AST 168.7 ± 144.2				
Chen et al. [15]	NA	NA	NA	NA	NA	NA
Gong et al. 2015 [17]	ALT 143.3 ± 82.1	ALT 157.1 ± 82.1	6.7 ± 1.2	7.4 ± 1.2	HBeAg(+) 100%	HbeAg(+) 100%
	AST 70.2 ± 33.7	AST 70.2 ± 33.75.3				
Xu et al. [20]	ALT 119.2 ± 118.1	108.4 ± 75.2	5.8 ± 1.2	5.7 ± 1.2	HBeAg(+) 67.9%	HBeAg(+) 68.2%

ALT: alanine aminotransferase; AST: aspartate aminotransferase; NA: not available; ^▲^*P* < 0.05; ^▲▲^*P* < 0.01.

**Table 3:** Subgroup analysis based on the method used for diagnosis of fatty liver.

Outcome or subgroup	Study	Participants	RR/WMD (weighted mean difference) 95% CI	*P*
48W biochemical response	5	325/530	0.864/(0.778, 0.960)	0.007
Histopathology	2	65/120	0.980/(0.720, 1.352)	0.934
Ultrasound B	3	260/410	0.842/(0.754, 0.940)	0.002
48W virological response	7	380/623	0.896/(0.811, 0.989)	0.030
Histopathology	4	120/213	1.002/(0.773, 1.298)	0.987
Ultrasound B	3	260/410	0.866/(0.782, 0.959)	0.006
48W serological response	4	152/296	0.900/(0.661, 1.226)	0.504
Histopathology	3	87/148	0.936/(0.654, 1.340)	0.718
Ultrasound B	1	65/148	0.828/(0.458, 1.498)	0.533

RR: risk ratio; CI: confidence interval.
